# Global Trophic Position Comparison of Two Dominant Mesopelagic Fish Families (Myctophidae, Stomiidae) Using Amino Acid Nitrogen Isotopic Analyses

**DOI:** 10.1371/journal.pone.0050133

**Published:** 2012-11-28

**Authors:** C. Anela Choy, Peter C. Davison, Jeffrey C. Drazen, Adrian Flynn, Elizabeth J. Gier, Joel C. Hoffman, Jennifer P. McClain-Counts, Todd W. Miller, Brian N. Popp, Steve W. Ross, Tracey T. Sutton

**Affiliations:** 1 University of Hawaii, Department of Oceanography, Honolulu, Hawaii, United States of America; 2 Scripps Institution of Oceanography at University of California, San Diego, California, United States of America; 3 The University of Queensland, School of Biomedical Sciences, Brisbane, Australia; 4 Commonwealth Scientific and Industrial Research Organization, Marine and Atmospheric Research, Hobart, Australia; 5 University of Hawaii, Department of Geology & Geophysics, Honolulu, Hawaii, United States of America; 6 U.S. Environmental Protection Agency Office of Research and Development, National Health and Environmental Effects Research Laboratory, Mid-Continent Ecology Division, Duluth, Minnesota, United States of America; 7 United States Geological Survey, Southeastern Ecological Science Center, Gainesville, Florida, United States of America; 8 Ehime University, Global Center of Excellence, Center for Marine Environmental Studies, Matsuyama, Japan; 9 University of North Carolina at Wilmington, Center for Marine Sciences, Wilmington, North Carolina, United States of America; 10 College of William & Mary, Virginia Institute of Marine Science, Gloucester Point, Virginia, United States of America; University of Hamburg, Germany

## Abstract

The δ^15^N values of organisms are commonly used across diverse ecosystems to estimate trophic position and infer trophic connectivity. We undertook a novel cross-basin comparison of trophic position in two ecologically well-characterized and different groups of dominant mid-water fish consumers using amino acid nitrogen isotope compositions. We found that trophic positions estimated from the δ^15^N values of individual amino acids are nearly uniform within both families of these fishes across five global regions despite great variability in bulk tissue δ^15^N values. Regional differences in the δ^15^N values of phenylalanine confirmed that bulk tissue δ^15^N values reflect region-specific water mass biogeochemistry controlling δ^15^N values at the base of the food web. Trophic positions calculated from amino acid isotopic analyses (AA-TP) for lanternfishes (family Myctophidae) (AA-TP ∼2.9) largely align with expectations from stomach content studies (TP ∼3.2), while AA-TPs for dragonfishes (family Stomiidae) (AA-TP ∼3.2) were lower than TPs derived from stomach content studies (TP∼4.1). We demonstrate that amino acid nitrogen isotope analysis can overcome shortcomings of bulk tissue isotope analysis across biogeochemically distinct systems to provide globally comparative information regarding marine food web structure.

## Introduction

Deep oceanic waters (offshore depths >∼200 m) constitute the largest habitat on the planet. Industrialized fishing has substantially reduced the biomass of large predatory fishes (e.g., tunas, billfishes, sharks) within these deep ocean ecosystems [1]. There is growing evidence that overharvesting of these top trophic level animals may ultimately affect the stability and resilience of marine food webs through changes in system structure and function (e.g., [2], [3]). Improved understanding of trophic structure and food web interactions at a time of changing climate dynamics is critical for anticipating future changes in exploited marine populations. Particularly important is the need for comparative evaluation of potential fishery impacts on a global scale across biogeochemically and ecologically diverse systems.

Large-scale marine trophodynamics have traditionally been derived from stomach content (SC) analyses and more recently using stable isotope and fatty acid analyses. However, synthesizing multiple SC and/or biochemical datasets to compare ecosystem function between different oceanic regions can be difficult and is infrequently done. For the first time, we utilize a promising and emergent tool, compound-specific nitrogen isotope analysis of individual amino acids (CSIA), to compare the trophic positions (TPs) of widespread pelagic micronekton fishes from five biogeochemically distinct global ecosystems: Tasman Sea, California (CA) Current, Gulf of Mexico (GOM), northern Mid-Atlantic Ridge (MAR), and the North Pacific Subtropical Gyre (NPSG) near Hawaii.

In pelagic ecosystems, micronekton (small fishes, squids, and crustaceans ∼2–20 cm in size) are a critical trophic link between primary producers and higher trophic level consumers (e.g., tunas, seabirds, marine mammals). Dragonfishes (family Stomiidae) are considered the most diverse and numerically important higher-trophic level predatory meso- and bathypelagic fish group, while lanternfishes (family Myctophidae) are commonly the dominant micronekton organisms in terms of biomass and abundance in mesopelagic ecosystems (e.g., [4], [5]), and are thought to be the primary prey of most dragonfishes (e.g., [6], [7]). Widespread distributions and high biomass levels coupled with extensive diel vertical migrations suggest that these fishes are important mediators in the transfer of organic carbon between trophic levels and through a large part of the water column [8], often including benthic communities at continental margins [9].

Carbon (C) and nitrogen (N) stable isotope (SI) techniques have been extensively used in aquatic and terrestrial ecosystems, complimenting SC analyses by delineating TPs and tracing energy/nutrient flows [10], [11]. The basic premise underlying these studies is that preferential incorporation of ^15^N and ^13^C in consumer tissues results in predictable ∼2.0–3.4‰ increases in δ^15^N values and ∼0.5–0.8‰ increases in δ^13^C values relative to their prey at each subsequent trophic level [12], [13]. Inferring trophic connectivity from SI data requires sampling across multiple TPs, often a considerable logistic challenge in deep ocean systems. Ecological interpretation of SI data is often complicated by the inability to constrain temporal and spatial variability in the isotopic compositions of primary producers at the food web base [14]. In marine ecosystems like the NPSG for example, primary producers can seasonally switch between N_2_-fixation and upwelled nitrate-based production [15]. The δ^15^N values for atmospheric N_2_ (δ^15^N = 0‰) and inorganic deep-water nitrate (δ^15^N = ∼5–7‰) sources are distinct (e.g., [16]), and these differences are reflected in a consumer’s N isotopic composition [17].

Compound-specific isotope analysis of individual amino acids (AAs) is a developing technique that overcomes many of the limitations of bulk SI analysis. Instead of attempting to concurrently sample organisms representing multiple TPs in a food web, the CSIA approach uses the δ^15^N values of AAs of a consumer to constrain food web baseline isotopic variability and estimate TPs [18]. Laboratory experiments by McClelland and Montoya [19] demonstrated that certain “source” AAs (after [20]) (e.g., phenylalanine, glycine) fractionate very little with trophic processing and are indicative of the isotopic composition of the food web base. Other “trophic” AAs (e.g., glutamic acid, alanine) involved in transamination and deamination reactions undergo significant enrichment in ^15^N (∼7‰ per trophic level) and are thus indicative of the fractional TP of the consumer [21]. Using this approach, consumer TP can be estimated using a reasonably well-established relationship between trophic and source AAs [18], providing valuable information that can be utilized by ecosystem modelers and managers alike.

Many previous studies have successfully combined bulk SI and CSIA datasets across diverse phyla to demonstrate the advantages of the CSIA approach over traditional SI analysis (e.g., [20] in tuna, [17] in marine copepods, [22] in elasmobranchs). However, no previous studies have applied this approach across global marine ecosystems, and few have provided comparative information from multiple TPs. In this study, we conducted the first cross-system trophic comparison of two dominant marine fish consumer groups with well-characterized and distinct TPs across five unique biogeochemical regions. Although the TPs of the two fish groups appear consistent between regions based on available SC analyses, considerable regional variability in bulk tissue δ^15^N (δ^15^N_bulk_) values exists. Our CSIA data demonstrate that regional biogeochemistry directly influences fish δ^15^N_bulk_ values and suggest that across five global oceanic regions lanternfishes and dragonfishes may not be separated by a whole trophic level, which has implications for the exploited status of large marine ecosystems.

## Materials and Methods

### Sample Collection and Preparation

**Figure 1:**
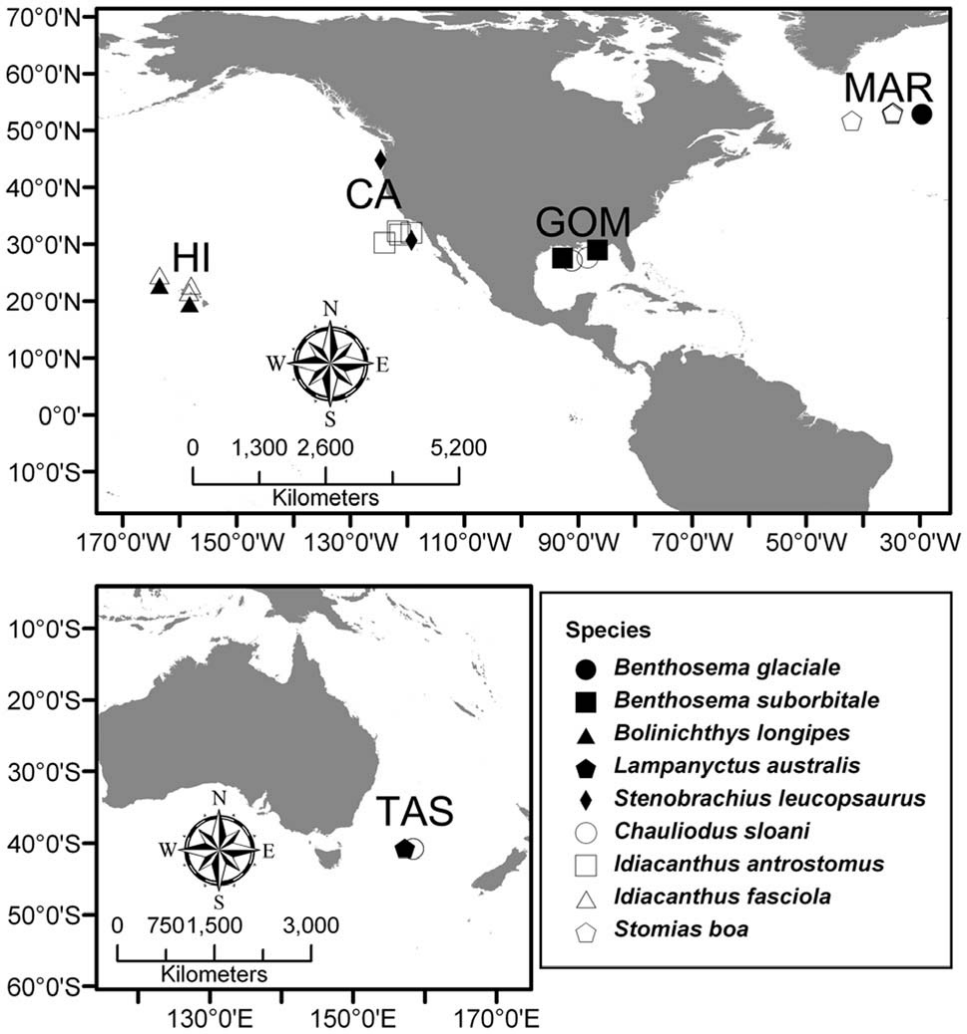
Map of sample collection locations. Approximate capture locations for species of lanternfish (closed symbols) and dragonfish (open symbols) specimens analyzed in this study, from five distinct and globally distributed regions (Tasman Sea (TAS), California Current (CA), Gulf of Mexico (GOM), Hawaii (HI), and the Mid-Atlantic Ridge (MAR)).

Fish specimens were independently collected during 2007–2011 by five research groups (one group per region) using a variety of midwater trawling equipment in five distinct regions (Figure 1; Table 1). For each region, fish species known to represent two distinct TPs (one species each of lanternfish and dragonfish) were carefully selected using existing SC data, were identified to the species level and measured (standard (SL) or total (TL) length), and frozen at sea until analysis (sample sizes in Table 1). Due to limited sample availability two dragonfish species were analyzed for the NPSG region, one of which was also sampled in the Tasman Sea and the GOM (Table 1). All species selected had region-specific SC data supporting the interpretation that the lanternfishes were zooplanktivorous (TP ∼3) and the dragonfishes were piscivorous (TP ∼4) (Table S1). In the laboratory, scales and skin were removed and white muscle tissue dissected from each specimen. Samples were oven-dried at ∼60°C for ∼48 hrs, ground and homogenized with a mortar and pestle, and shipped to the University of Hawaii (UH) for analysis. Tissue homogenates were split; splits were weighed and packaged into either tin capsules for bulk tissue SI analysis or combusted glass reaction vials for CSIA. This study was carried out in accordance with the animal use protocols of the University of Hawaii (protocol #10-984) and was approved by the UH Institutional Animal Care & Use Committee.

**Table 1:** Collection and size information of lanternfish (L) and dragonfish (D) specimens included in this study.

Region [Collection Year(s)]	Oceanographic Characterization	Species	Size Range Analyzed (mm)	Bulk δ^15^N (‰)	Bulk δ^13^C (‰)
**Hawaii (NPSG) [2010–2011]**	oligotrophic, subtropical	*Bolinichthys longipes* (L)	25–46 SL, n = 4 (a, b)	5.8±0.5	−18.6±0.7
		*Idiacanthus fasciola* (D)	72–275 TL, n = 4 (a, b)	6.9±1.7	−17.5±1.2
		*Chauliodus sloani* (D)	137 SL, n = 1 (a, b)	7.2	−17.6
**Tasman Sea Abyssal Basin [2008]**	subtropical convergence, temperate	*Lampanyctus australis* (L)	86–107 SL, n = 6 (a);86–103 SL, n = 3 (b)	11.3±1.0	−18.0±0.6
		*C. sloani* (D)	190–280 SL, n = 5 (a);255–280 SL, n = 2 (b)	11.0±0.9	−18.7±0.3
**Gulf of Mexico [2007]**	oligotrophic, subtropical	*Benthosema suborbitale* (L)	19–27 SL, n = 4 (a, b)	6.9±0.7	−18.9±0.6
		*C. sloani* (D)	27–105 TL, n = 5 (a);27–105 TL, n = 3 (b)	8.0±0.6	−19.0±1.0
**Northern Mid-Atlantic Ridge [2009]**	high productivity, temperate-subtropical	*Benthosema glaciale* (L)	33–71 TL, n = 12 (a);33–71, n = 5 (b)	9.9±1.2	−18.8±0.5
		*Stomias boa* (D)	126–168 TL, n = 9 (a);142–168 TL, n = 3 (b)	10.4±0.7	−18.2±0.4
**California Current [2009–2010]**	high productivity, upwelling, temperate	*Stenobrachius leucopsaurus* (L)	41–100 SL, n = 11 (a);58–100 SL, n = 4 (b)	13.5±0.9	−19.6±1.5
		*Idiacanthus antrostomus* (D)	153–490 SL, n = 5 (a);158–318 SL, n = 2 (b)	16.0±0.8	−18.1±0.4

Fish size ranges are reported as standard length (SL) or total length (TL) measurements; sample sizes are also provided for specimens included in bulk tissue isotopic analyses (a) and AA nitrogen isotope analyses (b). Bulk tissue δ^15^N and δ^13^C values are summarized as mean ± S.D.

### Bulk Tissue Stable Isotope Analysis

Bulk SI analyses were performed at UH using an isotope ratio mass spectrometer (Delta^Plus^XP) coupled to an elemental analyzer (ConFlo IV/Costech ECS 4010). Isotopic values are reported in conventional δ-notation relative to the international standards atmospheric N_2_ and V-PDB, for N and C respectively. Accuracy and precision were <0.2‰ and were calculated using in-house reference materials analyzed every 10 samples (glycine and a tuna tissue homogenate, extensively characterized using NIST certified reference materials and verified independently in other isotope laboratories). Bulk tissue C isotope (δ^13^C_bulk_) values were corrected for lipid contribution using isotope mass balance based on deep-sea fish [23]. Tissue mass was limiting for some samples from two regions so previously measured δ^15^N_bulk_ and δ^13^C_bulk_ values for all samples from these regions were used (CA Current, *n* = 6, lanternfishes; GOM, *n* = 10, both fish groups). SI analyses for the CA Current followed Nam et al. [24] and analyses for the GOM followed McClain-Counts [25]. Of these 16 samples, enough tissue was available from nine samples to determine good agreement between analyses conducted at UH and those at other laboratories (Figure S1).

### TPs from Bulk Stable N Isotope Data

TP is commonly estimated using δ^15^N_bulk_ values (TP_bulk_) of consumers and their prey (e.g., [26]). We estimated TP_bulk_ using the following equations and regional data from the literature (see Table S3):
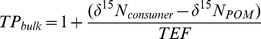
(1)

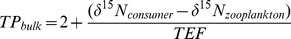
(2)


An average trophic enrichment factor (TEF) of 3‰ was used, a value within the range of reported variation amongst diverse organisms [13].

### Stable N Isotope Analysis of Individual AAs

A subset of fishes (33 of 66) was selected for CSIA based on broad ranges in size and δ^15^N_bulk_ values. Preparation for CSIA followed methods of Hannides et al. [17]. Dried samples were subjected to acid hydrolysis, esterification of the carboxyl terminus, and trifluroacetylation of the amine group [27]. Samples were redissolved in 50–100 µl of ethyl acetate, and the δ^15^N values of AAs were measured using an isotope ratio mass spectrometer (either a Delta^Plus^XP or Delta V Plus) interfaced with a gas chromatograph (Trace GC) through a GC-C III combustion furnace (980°C), reduction furnace (650°C) and liquid-N cold trap. Samples (1–2 µL) were injected (split/splitless injector, using a 10∶1 split ratio) onto a capillary column (BP×5 forte, 30 m×0.32 mm×1.0 µm film thickness) at an injector temperature of 180°C with a constant helium flow rate of 1.2 mL min^−1^. The column oven was held at 50°C for 2 min and then ramped to 190°C at a rate of 8°C min^−1^. At 190°C, temperature was increased to 300°C (at a rate of 10°C min^−1^) and held for 7.5 min. Samples were analyzed in triplicate, and the measured N isotopic compositions were normalized to known δ^15^N values of two co-injected internal reference compounds (norleucine and aminoadipic acid, δ^15^N reference values of 19.06‰ and −6.6‰ respectively). Reproducibility of isotopic analysis of glutamic acid and phenylalanine averaged ±0.5‰ (1 S.D.) and ranged from ±0.1‰ to ±2.4‰. Accuracy of the isotopic analysis was estimated using the known δ^15^N norleucine value to determine a measured δ^15^N value of aminoadipic acid, treating it as an unknown. Accuracy averaged ±1.3‰ (1 S.D.) and ranged from ±0.0‰ to ±3.5‰.

### TPs from CSIA Data

Chikaraishi et al. [18] measured the δ^15^N values of AAs in a variety of photoautotrophs and consumers and found that the relationship between glutamic acid (glu) and phenylalanine (phe) accurately described fractional TPs for a diversity of organisms:
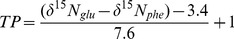
(3)


In equation (3), 3.4 is the difference between the δ^15^N values of glu and phe in marine primary producers (defined as β [18]), and 7.6 is the ^15^N TEF between glu and phe for each trophic level. Uncertainty resulting from AA-TP was calculated using propagation of errors by combining the uncertainty in β (±0.9‰) and TEF (±1.1‰) as determined by Chikaraishi et al. [18] and the measured analytical reproducibility of glu and phe δ^15^N values in each sample. Uncertainty in TP ranged from 0.1 to 0.9 (mean 0.3).

## Results

### Bulk Tissue Isotopic Analyses

δ^15^N_bulk_ values for both lanternfishes and dragonfishes differed significantly by region (ANOVA: *p*<0.001, F(4,33) = 76.07 for lanternfishes; *p*<0.001, F(4,24) = 74.41 for dragonfishes), and are reported in Table 1. δ^13^C_bulk_ values also differed significantly by region for lanternfishes (ANOVA: *p*<0.05, *F*(4,33) = 2.98) and dragonfishes (ANOVA: *p*<0.05, *F*(4,24) = 3.90). Temporal collection parameters were variable but relationships between individual fish δ^15^N_bulk_ and δ^13^C_bulk_ values and collection year were not significant and weak for δ^15^N_bulk_ (*p*>0.05, *r^2^* = 0.05), and δ^13^C_bulk_ values (*p*>>0.05, *r^2^* = 0.02). Individuals analyzed spanned a wide size range across the five regions (Table 1). Linear regressions of δ^15^N_bulk_ values on fish size per region and species groups were significant with negative slopes (*p*<0.05) for CA Current lanternfishes, and significant with positive slopes for MAR lanternfishes (*p*<0.05), but not significant for any other group and region pair (Figure S2). Comparison of δ^15^N_bulk_ and δ^13^C_bulk_ values indicated no significant differences between the two fish groups within a region (δ^15^N_bulk_: two-tailed paired t-test: *t* = 2.78, *p*>0.05; δ^13^C_bulk_: two-tailed paired t-test: *t* = 2.78, *p*>0.05). Estimates of TP_bulk_ were variable across the five regions for both lanternfishes and dragonfishes (ranges for individual specimens were TPs 1.8–4.5 and TPs 1.5–4.8, respectively) and did not align with TPs estimated from SC studies (Table S1, Table 2, Table S3).

**Table 2:** Regional comparison of lanternfish and dragonfish trophic positions estimated by amino acid and bulk tissue isotopic data.

Region	Mean LanternfishTP_bulk_ *	Mean DragonfishTP_bulk_*	TP_bulk_Difference	Mean LanternfishAA-TP#	Mean DragonfishAA-TP#	AA-TPDifference
**North Pacific Subtropical** **Gyre (Hawaii)**	2.0±0.1	2.3±0.5	0.3 TP	2.6±0.2	3.2±0.1	0.6 TP
**Tasman Sea**	2.7±0.3	2.6±0.3	0.1 TP	2.8±0.0	3.0±0.1	0.2 TP
**Gulf of Mexico**	2.0±0.2	2.4±0.2	0.4 TP	2.9±0.2	3.0±0.3	0.1 TP
**Mid-Atlantic Ridge**	4.1±0.4	4.3±0.2	0.2 TP	3.2±0.4	3.4±0.2	0.2 TP
**California Current**	3.4±0.3	4.2±0.3	1.2 TP	2.8±0.1	3.3±0.1	0.5 TP

# Calculated using Eq. 3.

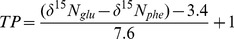
 (3)*Calculated using Eq. 1 and Eq. 2 as described in “Methods.” Region-specific δ15NPOM and δ15Nzooplankton values from the literature are presented in Table S3.

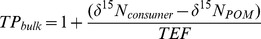
 (1)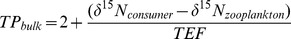 (2)

Summarized values include mean trophic positions (TPs) calculated from bulk tissue δ^15^N values (TP_bulk_) using a trophic enrichment factor (TEF) of 3‰ (mean ± S.D.), and TPs calculated from AA-CSIA data (AA-TP) (mean ± S.D.). Differences in the calculated means between dragonfishes and lanternfishes are shown. Dragonfish values for Hawaii include specimens of both *Chauliodus sloani* and *Idiacanthus fasciola*.

### Stable N Isotope Analysis of Amino Acids & TP Estimates

**Figure 2:**
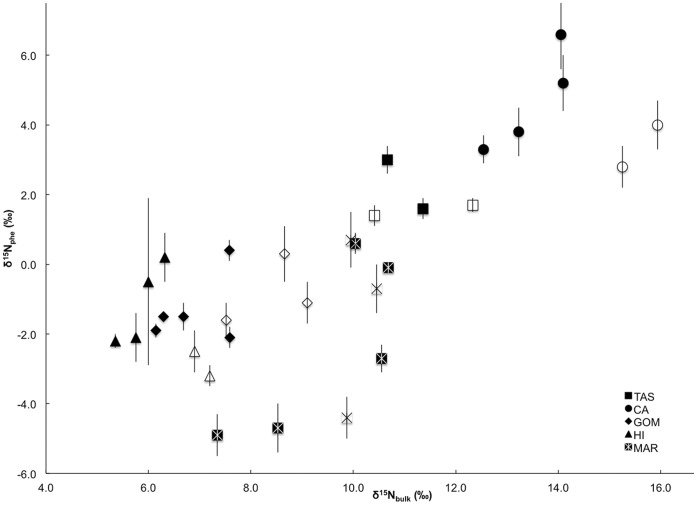
Influence of regional biogeochemistry on consumer isotopic composition. Relationship between δ^15^N values of phenylalanine (δ^15^N_phe_) (‰) and bulk white muscle tissue (δ^15^N_bulk_) (‰) in 33 specimens of mesopelagic lanternfishes (empty symbols) and dragonfishes (filled symbols) from offshore waters of the Tasman Sea (TAS), California Current (CA), Gulf of Mexico (GOM), Hawaii (HI), and the Mid-Atlantic Ridge (MAR). Error bars are standard deviations. δ^15^N_phe_ and δ^15^N_bulk_ values in fishes across all regions are significantly positively correlated (p<0.05, r^2^ = 0.58; y = 0.75× –7.14).

Similar to regional differences in δ^15^N_bulk_ values, variability in the δ^15^N values of the source AA phenylalanine (δ^15^N_phe_) was apparent (mean 0.0‰, range −4.9 to 6.6‰). Importantly, δ^15^N_phe_ values for both fish groups differed significantly by region (ANOVA, *p*<0.05). Conversely, the δ^15^N_phe_ values of lanternfishes and dragonfishes within a region were not significantly different (two-tailed paired t-test: *t* = 1.03, p>0.05). The significant correlation between δ^15^N_phe_ and δ^15^N_bulk_ values (Figure 2) suggested that δ^15^N_bulk_ values predominantly reflect the δ^15^N values of primary producers in each region (i.e., the regional isotopic baseline).

Variability in the δ^15^N values of the trophic AA glutamic acid (δ^15^N_glu_) mirrored regional patterns in δ^15^N_phe_ values (mean 18.6‰, range 12.1–24.1‰); the highest δ^15^N_glu_ values were observed in both fish groups from the productive regions of the CA Current, the Tasman Sea and the MAR (Table S2). Conversely, the lowest δ^15^N_glu_ values were observed in the fishes from the tropical oligotrophic waters of the GOM and the NPSG. Similar to regional differences in δ^15^N_phe_ values, differences in δ^15^N_glu_ values across regions were also significant (ANOVA, *p*<0.05).

**Figure 3:**
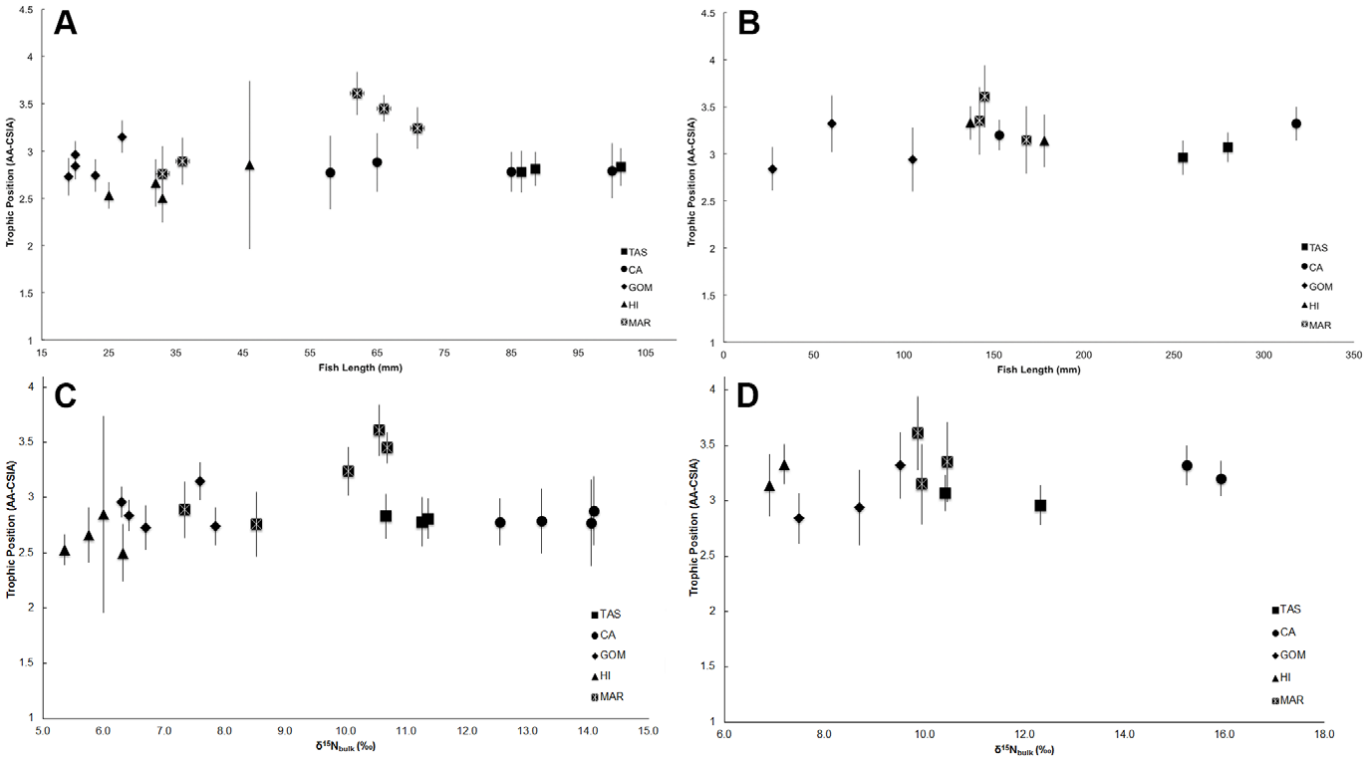
Uniform amino acid based trophic positions for lanternfishes and dragonfishes. Relationship between fish length (mm) and amino acid CSIA estimated trophic positions of a) individual lanternfishes, and b) individual dragonfishes from five regions. Also shown is the relationship between individual fish bulk tissue δ^15^N values (‰) and amino acid CSIA estimated trophic positions of c) lanternfishes and d) dragonfishes from five regions. Error bars indicate propagated error from trophic position calculation (see methods).

TPs calculated from CSIA data (AA-TPs) for lanternfishes and dragonfishes using eq. 1 were very consistent across the five regions despite great variability in δ^15^N_bulk_ and δ^15^N_phe_ values, as well as fish size (Figure 3). In all five regions the AA-TPs of dragonfishes were much lower than the expected TP of 4.1 based on SC studies. There was no statistical difference in mean dragonfish AA-TPs among regions (ANOVA, F = 1.62, *p*>0.05), indicating similar TPs across all ecosystems studied. Mean lanternfish AA-TPs were significantly different across the five regions (ANOVA, F = 4.16, *p*<0.05). However, this difference was primarily driven by MAR lanternfishes (mean AA-TP: 3.2), which had elevated AA-TPs relative to fishes from the other regions (mean AA-TPs range: 2.6 to 2.9). Mean lanternfish AA-TPs were significantly lower than mean dragonfish AA-TPs within a region (two-tailed paired t-test, *t* = 2.78, *p*<0.05).

## Discussion

Despite regional oceanographic influences on bulk SI and CSIA data, strongly uniform AA-TPs were observed across the five global regions for both fish groups. Significant differences in AA-TPs between the two fish groups were consistent with previous SC analyses, though different in magnitude. The biogeochemical diversity present in the five regions was reflected in both δ^15^N_bulk_ and δ^15^N_phe_ values (δ^15^N_phe_ = −4.9 to 6.6‰, Table S2), and is consistent with baseline N values produced by known biogeochemical processes (microbially-mediated N-recycling dynamics in the oligotrophic gyres to nitrate-based upwelling in the CA Current, for example). The strong correlation between δ^15^N_bulk_ and δ^15^N_phe_ consumer values indicates that regional biogeochemical differences influenced δ^15^N_bulk_ values in higher consumers (Figure 2).

The lack of comprehensive regional information characterizing isotopic baselines (i.e., encompassing uniform seasons and years) inhibits a more detailed and accurate calculation of TP from δ^15^N_bulk_ values across regions. TPs calculated from δ^15^N_bulk_ values were more variable across regions than TPs estimated from either SC studies or CSIA (Table S1, Table 2, Table S3). Absolute AA-TPs for lanternfishes were all within the range of those estimated by SC studies, while absolute AA-TPs for dragonfishes were lower than ranges derived from SC studies. AA-TPs also provided new information suggesting that these two fish groups plausibly have more similar TPs across the global oceans than previously documented by SC data. These results highlight the potential advantages of CSIA-based TPs for food web analysis in remote or highly heterogeneous ecosystems where baseline δ^15^N_bulk_ values are deficient or difficult to obtain.

One explanation for the apparent disagreement between dragonfish SC (∼TP 4.1) and AA-TP (∼TP 3.2) estimates is that the TEF used to establish TP is different for the two fish groups. Stark energetic differences could result in different protein turnover rates and potentially different TEFs. Growth and metabolism data for these fish groups are limited, but two studies found that some species of dragonfish have exceptionally low metabolic rates in the deep ocean, about tenfold lower than the more active, diel-vertically migrating lanternfishes [28], [29]. Preferential retention of ^15^N is dependent upon protein-containing meals, wherein animals assimilate a fraction of the protein (somatic growth) and catabolize and excrete the remainder (metabolism). Tissue turnover information for the two fish groups are not available, however sporadic feeding coupled with low locomotory abilities and low metabolic rates could result in slower protein turnover in dragonfishes relative to lanternfishes.

A second explanation that may reconcile the differences between SC- and AA-derived TP estimates is that available SC data failed to integrate the mean fish diets examined. Ecologists have long recognized that SC analyses represent only a “snap-shot” of what an animal has recently eaten [30]. Seasonality and ontogeny, as well as variation in prey abundances, can affect the prey documented in fish stomachs [31]. Calculations of TP from SC data require knowledge of the TPs of animals forming a consumer’s prey base, many of which may be poorly known or also estimated from SC data. Additionally, depending on the digestibility of a prey item, organisms with resistant hard parts may be over-represented in SC analysis, while easily digested soft-bodied prey items can be overlooked. As a result, these biases could alter the TP estimated from SC analysis alone.

Results from this study show that amino acid CSIA can be a useful tool for elucidating and comparing trophic structure, which can potentially be broadly transferred to other ecosystems and organisms. More field and laboratory testing are needed before CSIA can be used to accurately estimate TPs for organisms for which diet and trophic information may be limited or missing entirely. Comparison of SC and isotopic data highlights the need for caution when establishing TPs from SC analysis alone, or vice versa. AA-TPs integrate dietary inputs over a longer time scale than SC analysis and yield a quantitative TP across a variety of species and temporal and spatial scales that can be easily contrasted. However, SC studies accomplish what isotopic analyses cannot – taxonomic identification of prey items – and thus cannot and should not be replaced.

Heated debate and pronouncements about the exploited status of large marine ecosystems are often built upon marine fish species mean or fractional TP assignments [2], [32]. Thus, TP estimates have widespread implications for describing energy flow, as well as within mathematical models that aim to simulate these ecosystems. Small changes in prey TP estimates can result in substantial changes in the estimates of top predator production. Both lanternfishes and dragonfishes are globally important prey items for many commercially important fishes as well as sharks and marine mammals (e.g., [33], [34], [35]). Our novel application of CSIA to a global sample set suggests that SC-derived TP estimates (and by extension, food web analyses) will benefit from combining an integrated CSIA approach that overcomes the challenges of using δ^15^N_bulk_ values across distinct regions. While SC analysis is a vitally important tool for food web characterization, it may also lead to errors in TP estimation.

The large differences observed in fish δ^15^N_bulk_ values represent anywhere from two to three TPs for two fish groups that are less than one TP apart according to available SC data. In contrast to SC studies and despite δ^15^N_bulk_ and δ^13^C_bulk_ variability, results of CSIA indicate: a) uniform TPs within both fish groups across all five regions, and b) consistent TPs between both fish groups across all five regions (Figure 3). CSIA results indicate that inconsistencies in δ^15^N_bulk_ values result from regionally distinct baseline N isotopic compositions. Although we acknowledge that our results raise specific uncertainties regarding this emerging food web tool and interpretation of traditional (SC) TP estimates that merit further investigation, the uniformity in CSIA-based TPs across global regions demonstrates that this is a promising method to compare food webs among ecologically and biogeochemically diverse ecosystems.

## Supporting Information

Figure S1 **Intra-laboratory comparison of measured bulk tissue δ^15^N values.** Comparison of bulk tissue δ^15^N (a) and δ^13^C (b) values measured at the University of Hawaii and two outside laboratories (University of North Carolina Wilmington (n = 3) and Ehime University (n = 6)). Neither the slope nor the intercept is different from 1 and 0, respectively at the 95% confidence interval.
(TIF)

Figure S2 **Relationship between fish length and bulk tissue nitrogen isotopic values in fishes.** Bulk tissue δ^15^N values (‰) versus fish standard length (mm) in a) lanternfishes and b) dragonfishes from five regions (TAS = Tasman Sea, CA = California Current, GOM = Gulf of Mexico, HI = Hawaii, MAR = mid-Atlantic Ridge).
(TIF)

Table S1 **Meta-analysis of region-specific published stomach content studies for lanternfish and dragonfish diet.** Meta-analysis of region-specific published food items (at the taxonomic level of Order) for lanternfish (L) and dragonfish (D) species and trophic positions (mean ± S.E.) (as published and defined by the *FISHBASE* online database (Froese and Pauly 2012)). Additional primary references listed may not be included in FISHBASE and are specific to fishes analyzed from each region. Food item column headers are as follows: %C is percent copepods, %O is percent ostracods, %E is percent euphausiids, %A is percent amphipods, %F is percent fishes, and %Oth is percent other (includes pteropods, gastropods and other molluscs, debris, salps, unidentified decapoda remains, etc.).
(DOCX)

Table S2 **Regional values of source and trophic amino acids in lanternfish and dragonfish.** Comparison of isotopic compositions of the source amino acid phenylalanine (δ^15^N_phe_) and the trophic amino acid glutamic acid (δ^15^N_glu_) (mean ± S.D.) in lanternfishes and dragonfishes across all five oceanographic regions. Dragonfish values for Hawaii include specimens of both *Chauliodus sloani* and *Idiacanthus fasciola*.
(DOCX)

Table S3 **Best available region-specific bulk stable nitrogen isotope data used to estimate fish trophic positions.** Summary of best available bulk stable nitrogen isotopic baseline values used to calculate trophic positions of lanternfishes and dragonfishes (TP_bulk_). Isotopic data characterizing regional food web bases from the same seasons and years was not available, highlighting the need for a more reliable method for calculating TPs from these isotopic data.
(DOCX)

## References

[1] Sibert J, Hampton J, Kleiber P, Maunder M (2006) Biomass, size, and trophic status of top predators in the Pacific Ocean. Science 314: 1773–1776. 17170304 10.1126/science.1135347

[2] Pauly D, Christensen V, Dalsgaard J, Froese R, Torres Jr F (1998) Fishing down marine food webs. Science 279: 860–863. 9452385 10.1126/science.279.5352.860

[3] Casini M, Hjelm J, Molinero J, Lovgren J, Cardinale M, et al (2009) Trophic cascades promote threshold-like shifts in pelagic marine ecosystems. Proc Natl Acad Sci U S A 106: 197–202. 19109431 10.1073/pnas.0806649105 PMC2629246

[4] Brodeur RD, Yamamura O [eds.] (2005) Micronekton of the North Pacific. PICES Science Report No. 30. North Pacific Marine Science Organization, Sidney, BC.

[5] De Forest L, Drazen J (2009) The influence of a Hawaiian seamount on mesopelagic micronekton. Deep-Sea Res, Part I 56: 232–250.

[6] Sutton TT, Hopkins TL (1996) Species composition, abundance, and vertical distribution of the stomiid (Pisces: Stomiiformes) fish assemblage of the Gulf of Mexcio. Bull Mar Sci 59: 530–542.

[7] Sutton TT, Hopkins TL (1996) Trophic ecology of the stomiid (Pisces, Stomiidae) assemblage of the eastern Gulf of Mexico: strategies, selectivity and impact of a mesopelagic top predator group. Mar Biol 127: 179–192.

[8] Hidaka K, Kawaguchi K, Murakami M, Takahashi M (2001) Downward transport of organic carbon by diel migratory micronekton in the western equatorial Pacific: its quantitative and qualitative importance. Deep-Sea Res, Part I 48: 1923–1939.

[9] Gartner J, Sulak VKJ, Ross SW, Necaise A (2008) Persistent near-bottom aggregations of mesopelagic animals along the North Carolina and Virginia continental slopes. Mar Biol 153: 825–841.

[10] Peterson B, Fry B (1987) Stable isotopes in ecosystem studies. Annu Rev Ecol Syst 18: 293–320.

[11] Jennings S, Barnes C, Sweeting CJ, Polunin NVC (2008) Application of nitrogen stable isotope analysis in size-based marine food web and macroecological research. Rapid Commun Mass Spectrom 22: 1673–1680. 18438766 10.1002/rcm.3497

[12] DeNiro M, Epstein S (1981) Influence of diet on the distribution of nitrogen isotopes in animals. Geochim Cosmochim Acta 45: 341–351.

[13] Vanderklift M, Ponsard S (2003) Sources of variation in consumer-diet δ^15^N enrichment: a meta-analysis. Oecologia 136: 169–182. 12802678 10.1007/s00442-003-1270-z

[14] Post DM (2002) Using stable isotopes to estimate trophic position: models, methods, and assumptions. Ecology 83: 703–718.

[15] Dore J, Brum J, Tupas L, Karl D (2002) Seasonal and interannual variability in sources of nitrogen supporting export in the oligotrophic subtropical North Pacific Ocean. Limnol Oceanogr 47: 1595–1607.

[16] Wada E, Hattori A (1976) Natural abundance of ^15^N in particulate organic matter in the North Pacific Ocean. Geochim Cosmochim Acta 40: 249–251.

[17] Hannides CCS, Popp BN, Landry M, Graham BS (2009) Quantification of zooplankton trophic position in the North Pacific Subtropical Gyre using stable nitrogen isotopes. Limnol Oceanogr 54: 50–61.

[18] Chikaraishi Y, Ogawa N, Kashiyama Y, Takano Y, Suga H, et al (2009) Determination of aquatic food-web structure based on compound-specific nitrogen isotopic composition of amino acids. Limnol Oceanogr: Methods 7: 740–750.

[19] McClelland J, Montoya J (2002) Trophic relationships and the nitrogen isotopic composition of amino acids in phytoplankton. Ecology 83: 2173–2180.

[20] Popp BN, Graham BS, Olson R, Hannides CCS, Lott M, et al (2007) Insight into the trophic ecology of yellowfin tuna, *Thunnus albacares*, from compound-specific nitrogen isotope analysis of proteinaceous amino acids. In Dawson TD, Siegwolf RTW, editors. Stable isotopes as indicators of ecological change. Terrestrial Ecology Series. Elsevier. 173–190.

[21] Chikaraishi YY, Kashiyama Y, Ogawa N, Kitazato H, Ohkouchi N (2007) Metabolic control of amino acids in macroalgae and gastropods: implications for aquatic food web studies. Mar Ecol Progr Ser 342: 85–90.

[22] Dale JJ, Wallsgrove NJ, Popp BN, Holland KN (2011) Nursery habitat use and foraging ecology of the brown stingray, *Dasyatis lata*, determined from stomach content, bulk and amino acid stable isotope analysis. Mar Ecol Prog Ser 433: 221–236.

[23] Hoffman J, Sutton TT (2010) Lipid correction for carbon stable isotope analysis of deep-sea fishes. Deep-Sea Res, Part I 57: 956–964.

[24] Nam NT, Miller T, Huan N, Tang V, Omori K (2011) Integrating community structure and stable isotope analysis to assess a heavily exploited coastal marine ecosystem off Central Vietnam. Fish Res 110: 268–276.

[25] McClain-Counts J (2010) Trophic structure of midwater fishes over cold seeps in the North-central Gulf of Mexico. M.S. thesis. Univ North Carolina Wilmington.

[26] Vander Zanden M, Cabana G, Rasmussen J (1997) Comparing trophic position of freshwater fish calculated using stable nitrogen isotope ratios (δ^15^N) and literature dietary data. Can J Fish Aquat Sci 54: 1142–1158.

[27] Macko S, Uhle M, Engel M, Andrusevich V (1997) Stable nitrogen isotope analysis of amino acid enantiomers by gas chromatography combustion/isotope ratio mass spectrometry. Anal Chem 69: 926–929. 21639229 10.1021/ac960956l

[28] Torres J, Belman B, Childress JJ (1979) Oxygen consumption rates of midwater fishes as a function of depth of occurrence. Deep-Sea Res 26: 185–197.

[29] Childress JJ, Taylor S, Cailliet G, Price M (1980) Patterns of growth, energy utilization and reproduction in some meso- and bathypelagic fishes off southern California. Mar Biol 61: 27–40.

[30] Hyslop E (1980) Stomach contents analysis: a review of methods and their application. J Fish Biol 17: 411–429.

[31] Kawaguchi K, Mauchline J (1982) Biology of myctophid fishes (family Myctophidae) in the Rockall Trough, northeastern Atlantic Ocean. Biol Oceanogr 1: 337–373.

[32] Branch T, Watson R, Fulton E, Jennings S, McGilliard C, et al (2010) The trophic fingerprint of marine fisheries. Nature 468: 431–435. 21085178 10.1038/nature09528

[33] Rosecchi E, Tracey DM, Webber WR (1988) Diet of orange roughy, *Hoplostethus atlanticus* (Pisces: Trachichthyidae) on the Challenger Plateau, New Zealand. Mar Biol 99: 293–306.

[34] Pilling GM, Purves MG, Daw TM, Agnew DA, Xavier JC (2001) The stomach contents of Patagonian toothfish around South Georgia (South Atlantic). J Fish Biol 59: 1370–1384.

[35] Cherel Y, Duhamel G (2004) Antarctic jaws: cephalopod prey of sharks in Kerguelen waters. Deep-Sea Res, Part I 51: 17–31.

