# Supplementary figures and images for: Global Trophic Position Comparison of Two Dominant Mesopelagic Fish Families (Myctophidae, Stomiidae) Using Amino Acid Nitrogen Isotopic Analyses
Source: PLoS One. 2012 Nov 28;7(11):e50133. doi: 10.1371/journal.pone.0050133 (PMC3509156; doi:10.1371/journal.pone.0050133)

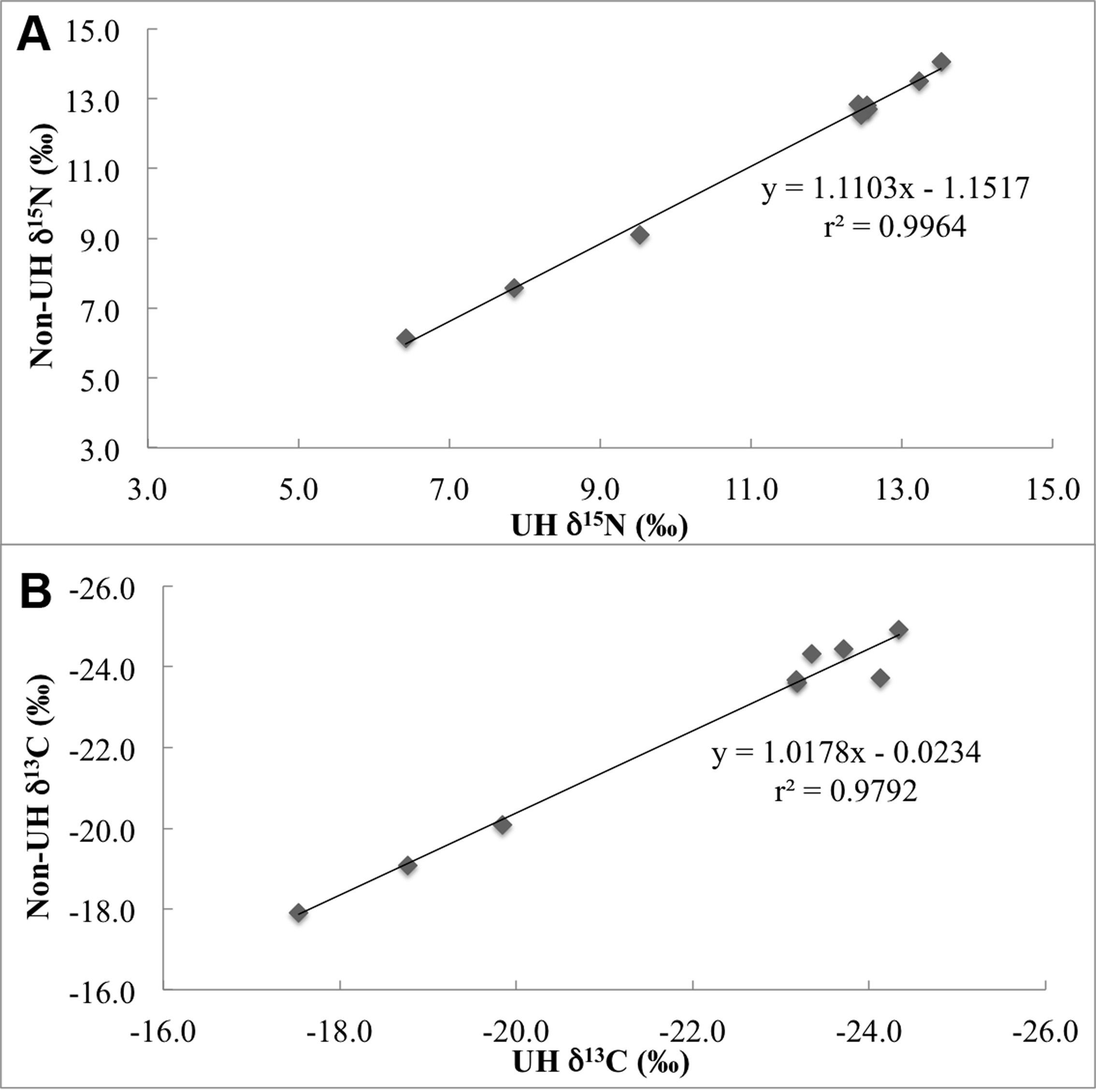

Supplement: Figure S1 — Intra-laboratory comparison of measured bulk tissue δ15N values. Comparison of bulk tissue δ15N (a) and δ13C (b) values measured at the University of Hawaii and two outside laboratories (University of North Carolina Wilmington (n = 3) and Ehime University (n = 6)). Neither the slope nor the intercept is different from 1 and 0, respectively at the 95% confidence interval. (TIF) [file pone.0050133.s001.tif]

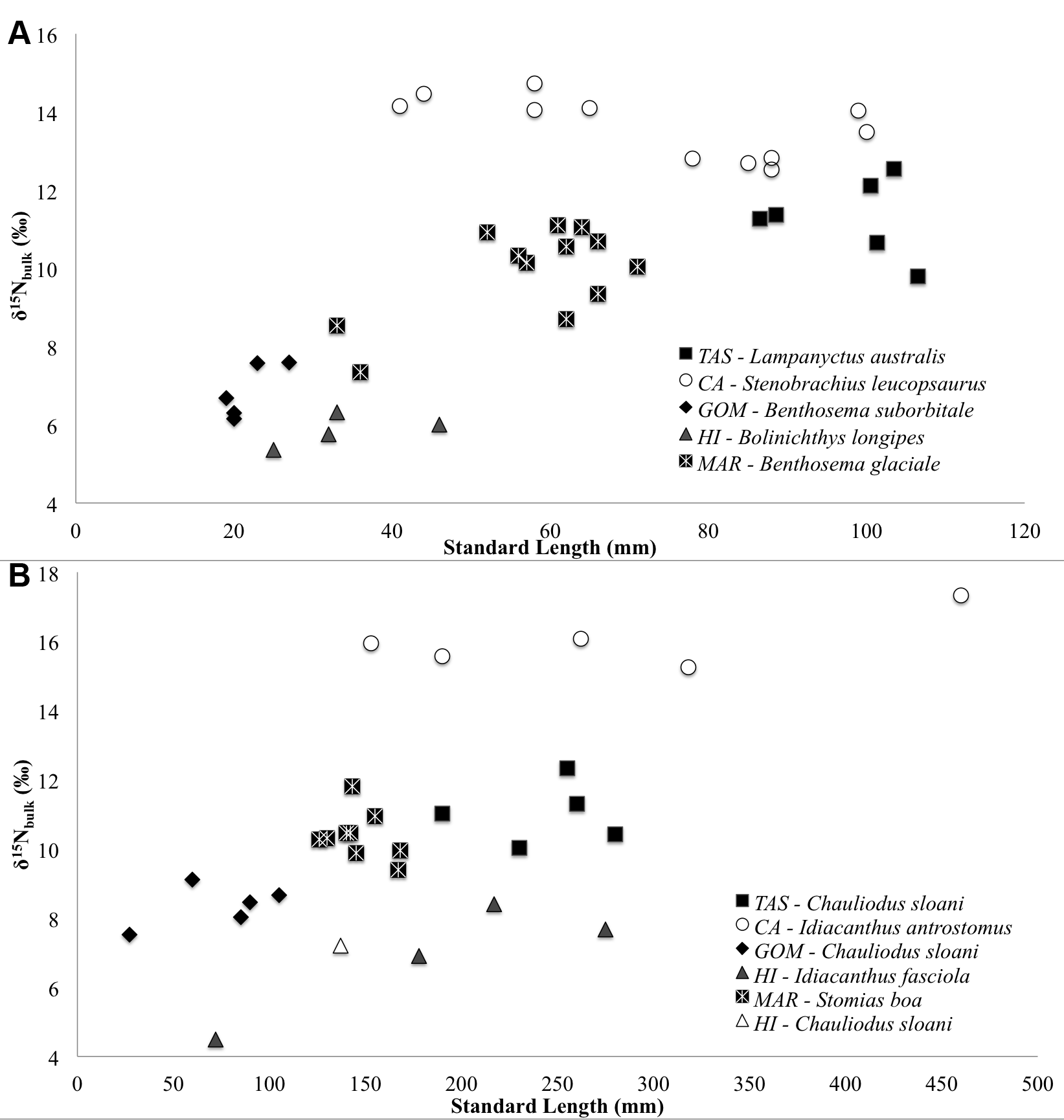

Supplement: Figure S2 — Relationship between fish length and bulk tissue nitrogen isotopic values in fishes. Bulk tissue δ15N values (‰) versus fish standard length (mm) in a) lanternfishes and b) dragonfishes from five regions (TAS = Tasman Sea, CA = California Current, GOM = Gulf of Mexico, HI = Hawaii, MAR = mid-Atlantic Ridge). (TIF) [file pone.0050133.s002.tif]
