# Supplementary material for: Global Trophic Position Comparison of Two Dominant Mesopelagic Fish Families (Myctophidae, Stomiidae) Using Amino Acid Nitrogen Isotopic Analyses
Source: PLoS One. 2012 Nov 28;7(11):e50133. doi: 10.1371/journal.pone.0050133 (PMC3509156; doi:10.1371/journal.pone.0050133)
Supplement: Table S1 — Meta-analysis of region-specific published stomach content studies for lanternfish and dragonfish diet. Meta-analysis of region-specific published food items (at the taxonomic level of Order) for lanternfish (L) and dragonfish (D) species and trophic positions (mean ± S.E.) (as published and defined by the FISHBASE online database (Froese and Pauly 2012)). Additional primary references listed may not be included in FISHBASE and are specific to fishes analyzed from each region. Food item column headers are as follows: %C is percent copepods, %O is percent ostracods, %E is percent euphausiids, %A is percent amphipods, %F is percent fishes, and %Oth is percent other (includes pteropods, gastropods and other molluscs, debris, salps, unidentified decapoda remains, etc.). (DOCX) [file pone.0050133.s003.docx]

**Table S1.**

| Region | Species | Meta-analysis of food items | | | | | | Number of published food item records | Primary References | FISHBASE Trophic Position* |
| --- | --- | --- | --- | --- | --- | --- | --- | --- | --- | --- |
|  |  | **% C** | **% O** | **% E** | **% A** | **% F** | **% Oth** |  |  |  |
| **North Pacific Subtropical Gyre (Hawaii)** | *Bolinichthys longipes* (L) | 78.1 | 9.4 | - | 3.1 | - | 9.4 | n = 32 | Clarke (1980) | 3.1 ± 0.22 |
|  | *Idiacanthus fasciola* (D) | - | - | - | - | 66.7 | 33.3 | n = 8 | Clarke (1982) | 3.92 ± 0.67 |
|  | *Chauliodus sloani* (D) | - | - | 15.8 | 5.3 | 57.9 | 21.0 | n=19 | Clarke (1982) | 4.20 ± 0.73 |
| **Tasman Sea Abyssal Basin** | *Lampanyctus australis* (L) | 14.3 | 7.1 | 14.3 | 14.3 | 14.3 | 35.8 | n=14 | Williams et al. (2001) | 3.25 ± 0.42 |
|  | *C. sloani* (D) | - | - | 15.8 | 5.3 | 57.9 | 21.0 | n=19 | Williams et al. (2001)  Butler et al. (2001) | 4.20 ± 0.73 |
| **Gulf of Mexico** | *Benthosema suborbitale* (L) | 52.6 | 13.2 | 6.6 | 7.9 | 1.3 | 18.4 | n = 76 | McClain-Counts (2010)  Hopkins et al. (1996)  Hopkins and Gartner (1992) | 3.40 ± 0.45 |
|  | *C. sloani* (D) | - | - | 15.8 | 5.3 | 57.9 | 21.0 | n=19 | Hopkins et al. (1996)  Sutton and Hopkins (1996) | 4.20 ± 0.73 |
| **Northern Mid-Atlantic Ridge** | *Benthosema glaciale* (L) | 33.3 | 9.1 | 21.2 | 12.1 | - | 21.2 | n=33 | Gjøsaeter (1973)  Kinzer (1977)  Roe and Badcock (1984)  Sameoto (1988) | 2.99 ± 0.29 |
|  | *Stomias boa* (D) | - | - | - | - | 80.0 | 20.0 | n = 5 | Mauchline and Gordon (1983)  Borodulina (1971) | 3.95 ± 0.64 |
| **California Current** | *Stenobrachius leucopsaurus* (L) | 55.8 | 3.4 | 11.7 | 10.2 | 2.4 | 16.5 | n = 206 | Beamish (1999)  Pearcy et al. (1979)  Suntsov and Brodeur (2008) | 3.23 ± 0.26 |
|  | *I. antrostomus* (D) | 0 | 0 | 0 | 0 | 100 | 0 | n=3 | Borodulina (1971) | 3.83 ± 0.60 |

**Literature Cited**

Beamish, R. J., K. D. Leask, O. A. Ivanov, A. A. Balanov, A. M. Orlov, and B. Sinclair. 1999. The ecology, distribution, and abundance of midwater fishes of the Subarctic Pacific gyres. Progress in Oceanography 43:399-442.

Borodulina, O.D. 1971. The feeding of mesopelagic predatory fish in the open ocean. Voprosy ikhtyologii 12:757-768.

Butler, M., S. M. Bollens, B. Burkhalter, L. P. Madin, and E. Horgan. 2001. Mesopelagic fishes of the Arabian Sea: distribution, abundance and diet of *Chauliodus pammelas*, *Chauliodus sloani*, *Stomias affinis* and *Stomias nebulosus*. Deep-Sea Research, Part II 48:1369-1383.

Clarke, T. A. 1980. Diets of fourteen species of vertically migrating mesopelagic fishes in Hawaiian waters. Fishery Bulletin 78:619-640.

Clarke, T. A. 1982. Feeding habits of stomiatoid fishes from Hawaiian waters. Fishery Bulletin 80:287-304.

Hopkins, T. L. and J. V. Gartner, Jr. 1992. Resource-partioning and predation impact of a low-latitude myctophid community. Marine Biology 114:185-197.

Gjøsaeter, J. 1973. The food of the myctophid fish, *Benthosema glaciale* (Reinhardt), from western Norway. Sarsia 52:53-58.

Hopkins, T. L., T. T. Sutton, and T. M. Lancraft. 1996. The trophic structure and predation impact of a low latitude midwater fish assemblage. Progress in Oceanography 38:205-239.

Kinzer, J. 1977. Observation on feeding habits of the mesopelagic fish *Benthosema glaciale* (Myctophidae) off NW Africa, p. 381-392. *In* Anderson, W. R., Zahuranec, B. J. [eds.], Oceanic sound scattering prediction. Plenum Press.

Mauchline, J. and J. D. M. Gordon. 1983. Diets of clupeoid, stomiatoid, and salmonoid fish off the Rockall Trough, northeastern Atlantic Ocean. Marine Biology 77:67-78.

McClain-Counts, J. P. 2010. Trophic structure of midwater fishes over cold seeps in the North-central Gulf of Mexico. M.S. thesis. Univ. North Carolina Wilmington.

Pearcy, W. G., H. V. Lorz, and W. Peterson. 1979. Comparison of the feeding habits of migratory and non-migratory *Stenobrachius leucopsarus* (Myctophidae). Marine Biology 51:1-8.

Roe, H. S. J. and J. Badcock. 1984. The diel migrations and distributions within a mesopelagic community in the north east Atlantic. 5. Vertical migrations and feeding of fish. Progress in Oceanography 13:389-424.

Sameoto, D. D. 1988. Feeding of lanternfish *Benthosema glaciale* off the Nova Scotia Shelf. Marine Ecology Progress Series 44:113-129.

Sunstov, A. V. and R. D. Brodeur. 2008. Trophic ecology of three dominant myctophid species in the northern California Current region. Marine Ecology Progress Series 373:81-96.

Sutton, T. T. and T. L. Hopkins. 1996. Species composition, abundance, and vertical distribution of the stomiid (Pisces: Stomiiformes) fish assemblage of the Gulf of Mexcio. Bulletin of Marine Sciences 59:530-542.

Williams, A., J. A. Koslow, A. Terauds, and K. Haskard. 2001. Feeding ecology of five fishes from the mid-slope micronekton community of southern Tasmania, Australia. Marine Biology 139:1177-1192.
