# Supplementary material for: Global Trophic Position Comparison of Two Dominant Mesopelagic Fish Families (Myctophidae, Stomiidae) Using Amino Acid Nitrogen Isotopic Analyses
Source: PLoS One. 2012 Nov 28;7(11):e50133. doi: 10.1371/journal.pone.0050133 (PMC3509156; doi:10.1371/journal.pone.0050133)
Supplement: Table S2 — Regional values of source and trophic amino acids in lanternfish and dragonfish. Comparison of isotopic compositions of the source amino acid phenylalanine (δ15Nphe) and the trophic amino acid glutamic acid (δ15Nglu) (mean ± S.D.) in lanternfishes and dragonfishes across all five oceanographic regions. Dragonfish values for Hawaii include specimens of both Chauliodus sloani and Idiacanthus fasciola. (DOCX) [file pone.0050133.s004.docx]

**Table S2.**

| REGION | Lanternfish δ^15^N_phe_ | Lanternfish δ^15^N_glu_ | Dragonfish δ^15^N_phe_ | Dragonfish δ^15^N_glu_ |
| --- | --- | --- | --- | --- |
| **North Pacific Subtropical Gyre (Hawaii)** | -1.2 ± 1.2 | 14.7 ± 1.8 | -2.9 ± 0.5 | 17.5 ± 0.6 |
| **Tasman Sea** | 2.6 ± 0.9 | 19.7 ± 0.9 | 1.6 ± 0.2 | 20.4 ± 0.4 |
| **Gulf of Mexico** | -1.3 ± 1.0 | 16.4 ± 1.1 | -0.8 ± 1.0 | 18.0 ± 2.1 |
| **Mid-Atlantic Ridge** | -2.4 ± 2.5 | 17.7 ± 4.8 | -1.5 ± 2.6 | 19.9 ± 1.0 |
| **California Current** | 4.7 ± 1.5 | 21.9 ± 1.6 | 3.4 ± 0.8 | 24.0 ± 0.2 |
