# Supplementary material for: Global Trophic Position Comparison of Two Dominant Mesopelagic Fish Families (Myctophidae, Stomiidae) Using Amino Acid Nitrogen Isotopic Analyses
Source: PLoS One. 2012 Nov 28;7(11):e50133. doi: 10.1371/journal.pone.0050133 (PMC3509156; doi:10.1371/journal.pone.0050133)
Supplement: Table S3 — Best available region-specific bulk stable nitrogen isotope data used to estimate fish trophic positions. Summary of best available bulk stable nitrogen isotopic baseline values used to calculate trophic positions of lanternfishes and dragonfishes (TPbulk). Isotopic data characterizing regional food web bases from the same seasons and years was not available, highlighting the need for a more reliable method for calculating TPs from these isotopic data. (DOCX) [file pone.0050133.s005.docx]

**Table S3.**

| Region | Bulk Isotopic Baseline [Reference(s)] |
| --- | --- |
| **North Pacific Subtropical Gyre (Hawaii)** | Bulk δ^15^N values of particulate nitrate (PN) at 150m for Summer (3.0 ± 1.1‰) and Winter (3.5±1.3‰) [HOT Program, <http://hahana.soest.hawaii.edu/hot/>] |
| **Tasman Sea Abyssal Basin** | Bulk δ^15^N values of particulate organic matter (POM) at 20-250 m for Autumn (6.1 ± 2.5‰) [Davenport and Bax 2002] |
| **Gulf of Mexico** | Bulk δ^15^N values of POM at surface for August 2007 (3.8±1.9‰) n=21 [McClain-Counts 2010] |
| **Northern Mid-Atlantic Ridge** | δ^15^N values of *Calanus finmarchicus* at 0-1000 m (3.5‰, Petursdottir et al. 2008), *Diacria* sp. and *Cymbulia peroni* at 50-360 m (2.9‰ and 3.4‰, respectively; J. Hoffman, unpubl.) |
| **California Current** | δ^15^N values of POM at surface for June and August, 2000 and 2002 combined (shelf = 6.5‰, slope 5.5‰) [Miller et al. 2010] |

**Literature Cited**

Davenport S. and N. Bax. 2002. A trophic study of a marine ecosystem off southeastern Australia using stable isotopes of carbon and nitrogen. Canadian Journal of Fisheries and Aquatic Sciences 59:514-530.

McClain-Counts, J. P. 2010. Trophic structure of midwater fishes over cold seeps in the North-central Gulf of Mexico. M.S. thesis. Univ. North Carolina Wilmington.

Miller, T. W., R. D. Brodeur, G. Rau, and K. Omori. 2010. Prey dominance shapes trophic structure of the northern California Current pelagic food web: evidence from stable isotopes and diet analysis. Marine Ecology Progress Series 420:15-26.
